# The risk of infections in hematologic patients treated with rituximab is not influenced by cumulative rituximab dosage - a single center experience

**DOI:** 10.1186/1471-2334-14-364

**Published:** 2014-07-03

**Authors:** Johanna C Nissen, Margit Hummel, Joachim Brade, Jens Kruth, Wolf-Karsten Hofmann, Dieter Buchheidt, Mark Reinwald

**Affiliations:** 1 Institute of Clinical Radiology and Nuclear Medicine, Mannheim University Hospital, Theodor-Kutzer-Ufer 1-3, Mannheim, Germany; 2 Department of Hematology and Oncology, Medical Faculty Mannheim, University of Heidelberg, Theodor-Kutzer-Ufer 1-3, Mannheim, Germany; 3 Department of Medical Statistics and Biomathematics, Medical Faculty Mannheim, University of Heidelberg, Theodor-Kutzer-Ufer 1-3, Mannheim, Germany

## Abstract

**Background:**

Rituximab, a monoclonal antibody directed against CD20, is approved for the treatment of CD20-positive B-cell Non-Hodgkin’s lymphoma and rheumatologic disorders. Due to its potent activity in depleting CD20-positive lymphocytes, the influence on opportunistic infections is still under discussion. Thus, we analyzed the impact of rituximab either as monotherapy or in combination with other chemotherapeutic regimens to elucidate its role in contributing to infectious complications.

**Methods:**

The records of consecutive patients (n = 125, 141 treatment episodes) treated with rituximab alone or in combination with chemotherapy and corticosteroids were analyzed retrospectively for the incidence, spectrum and outcome of infections during treatment and 6 months after the last course of rituximab. Univariate analysis of cofactors such as steroid medication, antiinfective prophylaxis, underlying disease and remission status were performed.

**Results:**

Altogether 80 therapy episodes were associated with infections, the median number of infections per patient being 1 (range 1–7). The number of infectious complications was significantly higher in patients receiving a combination of rituximab and chemotherapy compared to rituximab monotherapy (p < 0.001). There was no statistically significant difference regarding number of rituximab courses or cumulative rituximab dosage between episodes with and without infections, respectively.Mean cumulative prednisone dosage between the cohort with infections and the one without infections showed a trend towards higher dosage of prednisone in the patients with infections (mean difference 441 mg, p > 0.14).

**Conclusions:**

Rituximab in induction treatment, either as monotherapy or combined with chemotherapy by itself does not increase the incidence or change the spectrum of infections in hematologic patients. However the possible influence of higher dosages of concomitant steroid medication on frequency of infections suggests that a heightened awareness of the potential for infectious complications should be applied to patients receiving higher doses of glucocorticoids in combination with other therapeutic regimens.

## Background

Rituximab, a monoclonal antibody directed against the CD20 epitope, was approved in 1998 in Europe for treatment of CD20-positive B-cell non Hodgkin’s lymphoma. It has shown significant increase of survival in B-cell malignancies and has become standard of care in various entities of lymphomas and other malignant hematologic diseases. Recent data furthermore suggests an even better outcome for indolent B-cell malignancies if rituximab is continued after the end of the chemotherapeutic regimen as a maintenance treatment [1] for follicular lymphoma and for mantle cell lymphoma [2]. Due to its good activity in a variety of autoimmune diseases rituximab has been approved for the treatment of rheumatoid arthritis (RA) [3] and ANCA-associated vasculitis [4]. Beyond its approval, rituximab is being used and/or evaluated for further disease entities like immune thrombocytopenia [5], autoimmune hemolytic disease [6], posttransplant lymphoproliferative disorders [7] and multiple sclerosis [8].

Based on these data, the principle of anti-CD20-based monoclonal therapy has lead to research in more agents targeting CD20, namely Ofatumumab (Arzerra®), approved for chronic lymphocytic leukemia and more recently Obinutuzumab [9].

As CD20 is also expressed on healthy cells, there are concerns that the incidence of infections may increase: Treatment with rituximab leads to a pronounced depletion of pre-B-cells and mature-B-cells for several months, with levels returning to normal about 12 months after the last application. As CD20 is not expressed on healthy plasma cells, immunoglobulin levels were initially thought to be unaffected by rituximab treatment [10], recent data however, suggest an increased risk of hypogammaglobulinemia for patients during maintenance treatment [11]. Moreover, late-onset neutropenia after rituximab administration has been described repeatedly [12].The risk of infectious complications in patients receiving rituximab is still under discussion: Although some groups found an increase in infections [13] for NHL patients, others could not reproduce that finding [14] for NHL. A recent metanalysis covering three randomized controlled trials also failed to find an increase in infections in RA patients treated with rituximab [15]. However, judging the influence of rituximab on incidence of infection is difficult as this agent is often part of a complex treatment regimen consisting of different chemotherapeutic drugs with each having a specific immunosuppressive effect. Indeed, in a randomized, phase III study evaluating the effect of rituximab maintenance treatment, the rate of CTC grade 3 or 4 neutropenia and rate of infectious episodes were significantly increased [1]. In renal transplant patients treated with rituximab**,** Kamar et al. described that the addition of rituximab to anti-thymocyte-globulin was an independent predictive factor for infection-related death [16] and a recent study showed that allogeneic stem cell recipients treated with rituximab for reactivation of Ebstein-Bar-Virus (EBV) had a moderate, but statistically significant higher non-relapse mortality due to an increase in bacterial infections [17].

A recent finding that has been acknowledged and which lead to a black-box-warning of the FDA is the statistical increase in progressive multifocal leucencephalopathy (PML) caused by reactivation of the JC virus initially observed in NHL patients but also recognized in RA patients treated with rituximab. A retrospective analysis recently described a significant higher incidence of PML cases for rituximab-treated patients [18], although it has to be kept in mind that NHL patients by itself do carry an increased incidence of PML.

Taken together, the exact influence of rituximab on incidence of infections is still controversial, probably depending on concomitant immunosuppressive medication (e.g. chemotherapy) and underlying disease. However, studies focusing on infectious complications of rituximab therapy are rare. In order to elucidate on that topic we performed a monocentric retrospective analysis by analyzing consecutive hematologic patients treated with rituximab in the timeframe from 2000 – 2005 for a variety of diseases for incidence and spectrum of infections as well as other factors contributing to infections, thereby trying to elucidate the role of rituximab in contributing to infection.

## Methods

The study was performed retrospectively on a cohort of unselected consecutive pts treated in the Hematology Department of the University Hospital of Mannheim with rituximab +/− chemotherapy between the 1st of February 2000 and the 31st of January 2005. The patients’ records were analyzed for the incidence of fever +/− infections and other clinical factors during treatment and 6 months after the last course of rituximab. Written informed consent for data collection was obtained within the consent procedure for cancer treatment and specialized medical care. As the study was done to retrospectively investigate clinical data of patients treated solely at our institution in a scientific intent and data were obtained anonymized concurrently, an approval of the local ethics committee (Ethics Committee, Faculty of Medicine Mannheim) was not required according to the German Ethics Committees regulations [19]. The study was performed in compliance with the Declaration of Helsinki.

### Patient characteristics

The study was performed on a cohort of 125 pts (male: 69, female: 56) of a median age of 65 years (range 16–87 years) and 141 therapy episodes were analyzed. Underlying diseases, chemotherapeutic regimens and comorbidities are depicted in Table 1.

**Table 1:** Characteristics of patients

**Characteristics**	**(n = 141)**	**Maximum CTC grade 1&2 infection**	**Maximum CTC grade 3&4 infection**
Underlying disease			
Indolent NHL	80	4	33
CLL	4	1	0
Aggressive NHL	49	6	32
ALL	4	0	3
AIHA	3	0	1
ITP	1	0	0
Therapeutic regimens used	(n = 141)		
Rituximab monotherapy	23	1	3
R-CHOP	84	7	42
*R-CHOEP*	4	1	3
*R-FC*	6	2	4
*R-FCM*	2	0	0
*R-DHAP*	2	0	2
*R-GMALL*	7	0	5
*R-Bendamustin*	4	0	1
*R-IMVP16*	2	0	2
*R-Gemcitabin*	1	0	1
*R-other*	6	0	6
*Comorbidities*			
*Diabetes mellitus*	18		
*Immunoglobulin deficiency*	10		
*Asplenia*	7		
*COPD*	5		
*Chronic sinusitis*	3		

CTC: common toxicity criteria 4.0 of the National Institute of Health; NHL: non-Hodgkin lymphoma; CLL: chronic lymphocytic leukemia; ALL: acute lymphoblastic leukemia; AIHA: autoimmune hemolytic anemia; ITP: immunothrombocytopenic purpura, R-CHOP: Rituximab, cyclophosphamide, adriamycin, vincristine, prednisone; R-CHOEP: Rituximab, cyclophosphamide, adriamycin, vincristine, etoposide, prednisone; R-FC: Rituximab, fludarabine, cyclopsphamide; R-FCM: Rituximab, fludarabine, cyclopsphamide, mitoxantrone; R-DHAP: Rituximab, cytarabine, cisplatin, dexamethasone; R-GMALL: Rituximab in combination with the german GMALL induction; protocol for ALL-therapy; R-Bendamustin: rituximab and bendamustine; R-IMVP16: Rituximab, ifosfamide, etoposide, methotrexate; R-other: consisting of combination of other therapies such as R-DAHP + DB, R-CHOEP + R-BALL/BNHL, R-FCM + R-Bendamustin, R-FC + R-liposomal Doxorubicin, R-FCM + R-HAP, R-CHOP + R-FC + R-Bendamustine, COPD: chronic obstructive pulmonary disease.

### Documented items

Patients’ records were analyzed for white blood cell count (WBC) and differential WBC counts, grading of WBC was done according to common-toxicity criteria 4.0 (CTC, http://evs.nci.nih.gov/ftp1/CTCAE/About.html) of the national cancer institute (NCI). T-cell deficiency was defined as CD4 + −cell percentage <25% or <450/μL, CD8 + −percentage <19% or <250/μl. Hypogammaglobulinemia was defined as aɣ-Globulin <8.7%, immunoglobulin deficiency was defined as serum IgG < 7,0 g/L, serum IgA <0.7 g/L or serum IgM < 0.4 g/L. The underlying disease as well as its stage and remission status were documented. Relapse was defined as a reoccurrence of symptoms or manifestations after obtaining complete remission for > =2 months, progressive disease was defined as a reoccurrence of symptoms or manifestations after obtaining at least a partial remission. All prior therapies as well as cumulative rituximab doses and dosage were documented and analysed. Type, dosage and duration of concomitant medication with known influence on infections (e.g. steroid dosage expressed in prednisone-equivalent) were analyzed and calculated. Comorbidities known to influence the risk of infection (e.g. diabetes mellitus, chronic obstructive pulmonary disease (COPD)) were included into the analysis. Supportive therapeutic measures like application of Granulocyte-colony–stimulating factor (G-CSF) and antiinfectious prophylaxis with Trimethoprim/Sulfamethoxazole (TMP-SMX) were documented.

Events judged as infectious episodes had to either provide a microbiologically documented infection (e.g. positive cultures), radiomorphologic signs of infections (e.g. pulmonary infiltrates) or fever of unknown origin (FUO). FUO was defined as a febrile episode ≥ 38.5°C where no infectious agent or focus had been obtained in the routine screening program consisting of a minimum of microbiological cultures, chest x-ray and abdominal ultrasound and other reasons (e.g. drug-induced fever) could be clinically ruled out. Diarrhea was defined as being of infectious nature if no other cause could be identified (e.g. toxic, concomitant medication). Opportunistic infections were defined as viral or fungal infections caused by known opportunistic pathogens. All infections were graded according to the CTC of the NCI.

### Statistical analysis

Patients’ data was collected, anonymized and inserted in a database (Microsoft Excel 2010; Microsoft Software, Redmond, USA). Descriptive statistics were performed using SAS software release 8.2 (SAS Institute, Cary, USA).Statistical tests consisted of the student *t*-test as well as the chi-square test. P values of less than .05 were considered statistically significant. Where applicable, relative risk of infection was determined by calculating the odds ratio.

For multivariable analyses, a linear regression analysis was performed. All variable, who were found to yield p-values of less than 0.1 in univariable analysis entered the multivariable model: The multivariable analyses were carried out with the SAS soft-ware, release 8.2 (SAS Institute, Cary, NC, USA).

For all statistical analyses patients who developed one or more infections were compared to the patients who did not develop an infection in the observed time period and who acted as the control group.

## Results

In total, 141 treatment phases (=cases) of 125 patients were evaluated. During the treatment phase and follow up, altogether 80 therapies were associated with infections, the median number of infections per patient being 1 (range 1–7). 61 therapeutic phases were not associated with an infectious episode in the observed time period.

The total number of infections registered was 138, in 45 and 35 treatment courses one and more than one infection (range 2–7) was found, respectively. Altogether 23 opportunistic infections were recognized, in one case, two opportunistic infections were noted. Forty-nine episodes of infection developed during hospitalization, in 58 infectious episodes, hospitalization was clinically indicated and 31 infectious episodes could be treated in an outpatient setting. In 88/138 infectious episodes intravenous antimicrobials were applied, in 46 episodes oral or local treatment was sufficient. Grading of infections according to CTC criteria revealed infections to be typically severe with 11 episodes consisting of grade 4 infections, 104 episodes being grade 3 infections and only a few mild infections (grade 2 = 23; grade 1 = 0). There was no difference of incidence of infection with regards to age, gender or grading of infections.

During the observation period, altogether 17 patients died. The majority (n = 14) of patients died either due to progressive disease or complications while suffering from progression of the underlying malignancy. Three of these patients died from pneumonia while having progressive disease and one patient while being in remission died to a disseminated *Varicella zoster* virus (VZV) infection.

### Microbiological spectrum of infectious episodes

In 59% of all infectious episodes no causative infectious agent could be identified. In the rest of these episodes the majority of observed pathogens were of bacterial origin (23%), with virus (14%) and fungi (4%) being the minority of observed infectious pathogens. Table 2 depicts the microbial spectrum and the site of detection of the infectious organism.

**Table 2:** Types of infection and infectious pathogen observed

**Type/localization of infection**	**n**	**Infections CTC grade 1&2**	**Infections CTC grade 3&4**
FUO	**37**	0	37
Upper respiratory tract infections	**14**	3	11
Urinary tract infections	**16**	10	6
Pneumonia	**18**	0	18
Bronchitis	**10**	6	4
Herpes zoster	**10**	3	7
Herpes labialis	**5**	3	2
Sepsis/bacteremia	**10**	0	10
GI-tract infections	**6**	2	4
Mucocutaneus candidiasis	**2**	1	1
Erysipel	**2**	0	2
Sinusitis	**2**	0	2
Cerebral aspergillosis	**1**	0	1
Other	**4**	2	2
**Infectious agent**			
Viral infections	**19**		
*Varicella Zoster*	**10**	3	7
*Herpes simplex*	**6**	3	3
*Cytomegaly*	**1**	0	1
*Norovirus*	**2**	1	1
Fungal infections	**6**		
*Aspergillus spp.*	**2**	0	2
*Candida spp.*	**4**	1	3
Bacterial infections	**32**		
*Escherichia coli*	**6**	4	2
*Campylobacter*	**1**	0	1
*Enterobacter*	**1**	0	1
*Salmonella*	**1**	0	1
*Staphylococcus epidermidis*	**10**	2	8
*Enterococcus spp.*	**2**	0	2
*Clostridium difficile*	**1**	1	0
*Combined*#	**10**	4	6

FUO: Fever of unknow norigin; other: Conjunctivitis, orbital phlegmonia, spondylodiscitis; # = combined infections: *Klebsiella pneumonia, Proteus mirabilis, Pseudomonas aeruginosa; Strepococcus salivarius, Streptococcus mitis oralis, Corynebacteria spp., Staphylococcus aureus.*

### Time of infection

**Figure 1:**
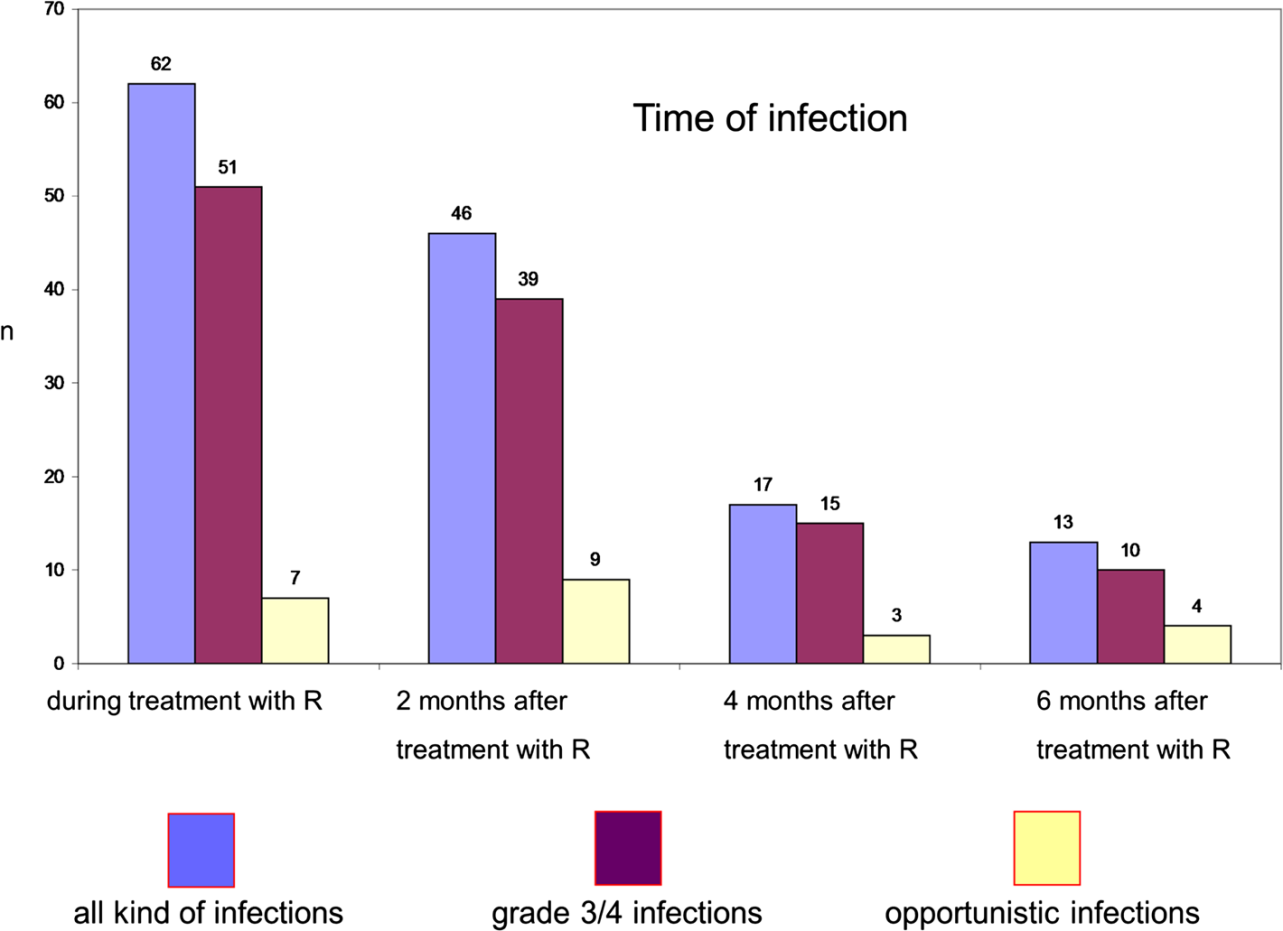
Infection relative to start of treatment 1 with Rituximab.

Median time of infection was 99 days after first administration of rituximab (range 1 – 566 days) (Figure 1). Rate of infections decreased with increasing distance to last application of therapy.

### Relationship of incidence of infection to other assessed factors

The combination of chemotherapy with rituximab lead to infections in 76/118 cases (64%), while rituximab monotherapy lead to infectious complications in only 4/23 cases (n = 17%). The number of infectious complications was significantly higher in patients receiving a combination of rituximab and chemotherapy compared to rituximab monotherapy (p < 0.001).

### Influence of chemotherapeutic regimen on incidence of infection

For patients receiving R-CHOP or R-CHOEP regimen, we identified 27 cases of infection (51%) for patients receiving R-CHOP/R-CHOEP therapy every 21 days (R-CHOP-21, R-CHOEP-21). For patients with intensified treatment consisting of R-CHOP or R-CHOEP every 14 days (R-CHOP-14 or R-CHOEP-14), the rate of infectious episodes was 26/35 episodes (74%). There was a significant increase of the rate of infection for patients receiving time-intensified R-CHOP/R-CHOEP every 14 days compared to the 21-days regimen (p < 0.03). Table 3 depicts the chemotherapeutic regimen and the identified focus of the infectious organism.

**Table 3:** Regimen, spectrum and type of infection

	**Cases with infections**	**Number of infectious episodes**	**Focus of infection**	**Opportunistic infections**	**Identified pathogen**	Number of CTC grade 3 + 4 infections*
**Rituximab monotherapy (n = 23)**	4	4	Pneumonia (n = 1)	2	none (n = 2)	3
					viral (n = 2)	
			Opp. Inf. (n = 2)			
			Bronchitis (n = 1)			
**R-CHOP/R-CHOEP (n = 88)**	53	80	UTI (n = 12)	15	none (n = 47)	66
					bacterial (n = 17)	
			URTI (n = 11)			
			Opp. inf. (n = 12)		viral (n = 15)	
			Opp. Pneumonia (n =5)		fungal (n = 3)	
			Bronchitis (n = 5)			
			FUO (n = 22)			
			Sepsis (n = 2)			
**R-FC/R-FCM (n = 8)**	6	9	Bronchitis (n = 3)	2	none (n = 4)	4
					bacterial (n = 3)	
			OppInf (n = 2)			
					viral (n = 2)	
			UTI (n = 1)			
			FUO (n = 2)			
			Sepsis (n = 1)			
**R-GMALL/R-DHAP (n = 9)**	7	19	UTI (n = 2)	2	none (n = 12)	16
					bacterial (n = 5)	
			Pneumonia (n = 2)			
					fungal (n = 2)	
			FUO (n = 8)			
			Sepsis (n = 4)			
			URTI (n = 1)			
			Opp Pneumonia (n = 1)			
			Sinusitis (n = 1)			
**R-Gemcitabine (n = 1)**	1	1	Pneumonia (n = 1)	none	0	1
**R-IMVP16 (n = 2)**	2	3	FUO (n = 1)	1	none (n = 2)	3
					viral (n = 1)	
			URTI (n = 1)			
			OppInf (n = 1)			
**R-Bendamustine (n = 4)**	1	3	Pneumonia (n = 1)	None	none (n = 1)	3
			Sepsis (n = 1)		bacterial (n = 2)	
			UTI (n = 1)			
**R-Other (n = 6)**	6	19	FUO (n = 4)	1	none (n = 13)	19
			Norovirus (n = 1)		viral (n = 1)	
			Diarrhea (n = 3)		bacterial (n = 4)	
			Pneumonia (n = 4)		fungal (n = 1)	
			Sepsis (n = 3)			
			Bronchitis (n = 1)			
			URTI (n = 1)			
			Sinusitis (n = 1)			
			Opp. Inf. (n =1)			

UTI = urinary tract infection, URTI = upper respiratory tract infection, FUO = fever of unknown origin, Opp = Opportunistic.

*in 35 cases more than one infection occured.

### Influence of rituximab dosage or number of doses on incidence or spectrum of infections

All patients were analyzed for the number of doses of rituximab and cumulative rituximab dosage. Rituximab standard dosage per course consisted of 375 mg/sqm, no dose escalation had been performed. Treatment courses associated with infections were compared with those without an infection during the observation period. Of the 80 treatment doses with infections, the number of administered rituximab doses was 6 and cumulative dosage was 3600 mg, whereas it was 5 doses and a cumulative dosage of 3500 mg for the patients without infections. There was no statistically significant difference in either number of doses or cumulative rituximab dosage when comparing courses with infectious complications (n = 80) compared to those without infections (n = 61) (p > 0.77).

### Influence of concomitant steroid medication on incidence or spectrum of infections

Median cumulative prednisone-dosage of all patients with infectious complications was 3500 mg prednisone while median cumulative prednisone-dosage of all those without infection was only1800 mg. When comparing cases with infectious complications (n = 80) compared to those without infections (n = 61) we found a significant higher amount of prednisone as concomitant medication for those with infections (p < 0.0003).

However, when performing a subgroup analysis of only those cases with rituximab in combination with chemotherapy (n = 118) (thus leaving out patients with rituximab monotherapy to avoid a potential bias), this significance was lost, although a trend towards higher mean cumulative prednisolone dosage in patients with rituximab-chemotherapy and infectious complications could still be observed (3220 mg vs 2778 mg, p > 0.14).

### Influence of WBC/differential blood cell count disease on incidence or spectrum of infection

Seventy-four cases who developed neutropenia during antineoplastic therapy were identified. Of those, 55/74 (74%) developed an infectious episode, one patient developed neutropenia while receiving rituximab monotherapy. Contrarily, treatment cases who did not develop neutropenia only suffered from an infectious complication in 37% (25/67).

Patients with infections suffered significantly more often from neutropenia compared to those without infections (p < 0.001).

### Age and comorbidity

When comparing cases with infectious complications (n = 80) compared to those without infections (n = 61) no statistical significant difference was identified for age (p > 0.69). When assessing the influence of comorbidities known to predispose for infections we found no significant difference for the following comorbidities: Diabetes, immunoglobulin deficiency, asplenia, chronic obstructive pulmonary disease and chronic sinusitis (p > 0.63).

### Supportive therapy and antiinfective prophylaxis

Altogether 63 cases received G-CSF after chemo-immunotherapy in the corresponding treatment phases; of those, 20 did not develop an infectious episode while the remaining 43 cases were associated with an infectious complication. Of cases developing infections (n = 80), 43 (54%) had been administered G-CSF in a prophylactic intention, whereas, only 20 cases (33%) of the patients without infections had received G-CSF.

Of those cases receiving TMP-SMX (n = 52), 42 (80%) developed an infectious complication, while 10 (19%) remained without infectious complications. Intravenous immunoglobulins were administered in 7/141 cases due to known hypogammaglobulinemia, 4 of these developed an infection.

### Statistical influential variables

When comparing the cases in which an infection developed (n = 80) with those without infections (n = 61) nominally scaled variables were analyzed and listed in Table 4.

**Table 4:** Statistical influential variables

**Variable**	**P-value (infections vs. No infection)**	**Odds ratio (95%CI)**
**Gender**	0,43	0,76 (0.39-1.49)
**Age (≤60,<60 years)**	0,69	1.15 (0.57-2.35)
**Stage**	0,076	n.a
**Number of prior therapies**	0,015*	n.a.
**Number of doses of Rituximab**	0,35	n.a.
**Chemotherapy in addition to Rituximab**	0,006*	n.a.
**Asplenia**	0,45	0.56 (0.12-2.58)
**Active malignancy at start of therapy**^**§**^	0,007*	6.75 (1.4-32.5)*
**Active malignancy at end of therapy**	0,02*	3.06 (1.14-8.17)*
**Application of G-CSF**	0,013*^#^	2.38 (1.19-4.76)
**Cotrimoxazole prophylaxis**	0,0001*^#^	5.64 (2.51-12.64)
**HIB/pneumococci vaccination**	0,09	0.18 (0.02-1.66)
**Substitution of immunoglobulins**	0,45	0.56 (0.12-2.58)
**Neutropenia**	0,0001*	4.86 (2.37-9.99)*
**Lymphopenia**	0,045*	6.96 (0.79-61.26)*
**Leukopenia**	0,0001*	4.51 (2.09-9.74)*
**Relevant comorbidities**	0,63	1.16 (0.54-2.49)

*statistically significant in univariate analysis.

n.a.: not applicable.

^§^ as patients with maintenance therapy and nonmalignant disorders had been treated with Rituximab not all patients had active malignancy at start of therapy.

^#^Paradoxically, patients with infectious complications had significantly more often been treated with G-CSF and received Cotrimoxazole prophylaxis, probably as these antiinfective measures had been administered to patients receiving more intensive chemotherapeutic regimens (such as GMALL, or R-CHOEP).

For the ordinally scaled variables assessed using the means and comparing the group with infections compared to those without infectious complications in this analysis the following results were found using a two-sided *t*-test:

There was no statistically significant difference regarding number of rituximab courses, cumulative rituximab dosage or patients’ age in the two groups with and without infections, respectively.

## Discussion

To investigate the incidence and spectrum of infections during and after rituximab treatment and evaluate other confounding variables which might contribute to the development of infectious complications, we performed this monocentric study.

For the interpretation of our study, it has to be kept in mind that it was done prior to wide-spread use of rituximab maintenance therapy which maximizes the amount of rituximab exposure and which makes evaluating the immunosuppressive effect of rituximab itself much easier as the confounding effect of the coadministered chemotherapeutic regimen is not present. As rituximab is an integral part of treatment regimens in certain hematologic disease groups, a direct comparison between treatment groups with and without rituximab is not possible. We therefore could only compare the incidence of infections during rituximab to historic controls without rituximab. Additionally, we analyzed all patients treated with rituximab and compared those with infectious complications to the ones without, trying to analyze in depth which factors contribute to these complications.

The overall incidence of infections in our study population is comparable to corresponding patient groups who received similar chemotherapy without rituximab. In the main patient group, i.e. patients receiving R-CHOP or R-CHOEP, the infection rate was higher in the group with a 14 day treatment interval as compared to the group with the 21 day interval. This is in accordance with other studies [20-22].

The spectrum of infections and pathogens is comparable to those reported in other studies, apart from one patient who had proven cerebral aspergillosis during R-CHOP treatment for NHL. Invasive fungal disease has so far been only reported with rituximab in combination with more intensive chemotherapy or after transplantation [23]. Whether rituximab increases the risk for invasive fungal disease cannot be stated from our data and will have to be investigated in larger patient numbers.

Similar as was recently presented by Lanini et al. [14] we could not detect a difference in rituximab dosage between those presenting with infection and the ones without, which is in line with the report mentioned above, suggesting, that, at least when combined with chemotherapy the influence of rituximab as an additional factor for infections is negligible. However, that data has to be interpreted with care, as the follow-up of our study was 6 months after end of therapy. Indeed in one of the major studies comparing the R-CHOP with the CHOP regimen for treatment of aggressive NHL [24] a trend for late infections was observed for the rituximab-containing regimen and could be at least partially attributed to increasingly recognized late-onset neutropenia induced by rituximab [25]. Another study for chronic lymphocytic leukemia patients evaluated the effect of ritxuximab when added to a regimen consisting of fludarabine and cyclophosphamide (FC); despite a longer follow up of 12 months, they could not find an increase in infections [26].

Recent data analyzing maintenance therapy however, show a significant increase in grade 3 and 4 infections for lymphoma patients receiving rituximab maintenance [1] compared to patients under observation, underlining that there is a potential for infections complications when receiving constant, steady dosages of rituximab, which prevents a regeneration of B-cell levels: The mean cumulative dosage of rituximab in our study was 3760 mg, which is much lower than the cumulative dosage of patients receiving induction and maintenance therapy with rituximab: In the study presented by Van Oers’, patients received 14 doses of rituximab treatment which accounts for at least 5250 mg/sqm per patient and which is nearly thrice the cumulative rituximab dosage of the patients studied in our trial, which might explain the different effects on infection incidence. Furthermore, the immunosuppressive effect of rituximab might be negligible compared to the pronounced effect of neutropenia-inducing chemotherapeutic regimens, but might become more apparent when compared with observation in maintenance therapy.

Taken together, our data suggest that the addition of rituximab does not increase the risk of infectious complications substantially at least during combined chemo-immunotherapy though its effect in maintenance therapy cannot be determined as our study only contained 6 patients during maintenance therapy. Though rituximab did not have an effect regarding the incidence of infections in our patient cohort, we possibly identified the cumulative steroid dosage to have a potential influence on the incidence of infections.

Evaluating the effect of steroids on infectious complications in chemotherapy patients is difficult as the majority of chemotherapeutic regimens contain glucocorticoids (e.g. CHOP, DHAP, Dexa-BEAM, etc.) and at least to our knowledge no randomized trial directly comparing a steroid-containing regimen to glucocorticoid-free regimen are published. For other hematologic diseases, data from myeloma trials elucidate the pronounced influence of high-dosed glucocorticoid medication on infectious complications: A recent monocentre experience suggests for patients receiving a combination of vincristine and adriamycin +/− intermittent high-dose dexamethasone that there is a significant decrease in rate of infections when abstaining from use of concomitant steroids [27]. In a multicenter trial evaluating the effect of high-dose dexamethasone versus low-dose dexamethasone in combination with lenalidomide for first-line myeloma treatment there was a significant increase in infectious complications for patients in the high-dose steroid group, underlining the influence of steroid dosage when added to another potentially immunosuppressive therapeutic regimen [28]. Especially for opportunistic infectious pathogens like *Aspergillus* spp., high dose steroids are a major independent factor for invasive aspergillosis and also confer an inferior prognosis in hematologic patients after allogeneic transplantation [29]. Furthermore, they are an independent risk factor for IA even for otherwise non-immunocompromised patients [30]: In a recent analysis [31] the majority of hematologic patients treated with rituximab with *Pneumocystis jirovecii* pneumonia had high-dose glucocorticoid exposure, underlining the pronounced effect of steroid co-medication in the pathogenesis of opportunistic infections.

Patients having infections in our study had a trend towards higher cumulative steroid dosages, however this effect was clearly less pronounced and lost its statistical significance when leaving out patients receiving rituximab monotherapy, possibly due to the lower amount of prednisolone in that less intensive, regimen.

A result in our study, which might seem contradictory at first, was that administration of G-CSF or TMP-SMX prophylaxis was present more frequently in the cohort with infectious complications. A possible explanation is that, as this was a retrospective, real-life observation, prophylaxis was not mandatory in patients; only 37% of all patients received TMP-SMX prophylaxis and 45% were administered G-CSF. This seemingly paradoxical result can be explained by the fact that patients with either a more intensive chemotherapeutic regimen (e.g. R-CHOP-14, R-DHAP, R-GMALL) or a regimen known for having more infectious complications (R-FC, R-FCM) received TMP-SMX prophylaxis and/or G-CSF, whereas those with less intensive regimens (e.g. R-CHOP21) did not. Indeed, though only a minor number of patients received R-FC or R-FCM and all were administered prophylaxis, we could detect a higher incidence of all infections in R-FC/R-FCM compared to patients with R-CHOP/R-CHOEP despite these prophylactic measures, however CTC grade 3/4 and thus severe infections were similar between R-FC/R-FCM and R-CHOP/R-CHOEP.

Our study has several limitations that need to be addressed: First, due to the retrospective, “real-life” design of our study, as we sought to identify factors discriminating patients with infectious complications from those without, we did not perform pair-matched analyses. Therefore it cannot be ruled out that there are other confounding factors which discriminate the groups and possibly influencing the results. Second, the late infections increasingly reported might have been missed as the follow-up was only 6 months. Third, as the trial ended in 2005, only a small minority of patients (n = 6) were already receiving rituximab maintenance treatment so the effect of rituximab maintenance treatment on incidence or spectrum of infections cannot be assessed from our data. Finally, another drawback of our study is that the study population is rather heterogeneous, regarding the underlying disease and the applied chemotherapy regimen. Preferably, a more homogeneous study population should be assessed.

## Conclusions

In summary, our data suggests that rituximab in induction treatment, either as monotherapy or combined with chemotherapy, by itself does not increase the incidence or change the spectrum of infections in hematologic patients. However a potential influence of higher dosages of concomitant steroid medication on frequency of infections may be present suggesting that a heightened awareness of the potential for infectious complications should be applied to patients receiving higher doses of glucocorticoids in combination with other therapeutic regimens.

## Competing interests

MR received a travel grant from Roche. All other authors declare that they have no competing interest.

## Authors’ contributions

JCN and MH conceived the study, developed the initial study protocol, with input from DB. JCN, MH, JK, MR and DB acquired and analyzed the data and coordinated the study. JB performed the statistical analysis and participated in writing of the manuscript. DB, WKH, JK and MR wrote the manuscript. All authors read and approved the final manuscript.

## Pre-publication history

The pre-publication history for this paper can be accessed here:

http://www.biomedcentral.com/1471-2334/14/364/prepub
